# Stable levels of *Coxiella burnetii* prevalence in dairy sheep flocks but changes in genotype distribution after a 10-year period in northern Spain

**DOI:** 10.1186/s13028-018-0429-x

**Published:** 2018-11-20

**Authors:** Raquel Álvarez-Alonso, Jesús Felix Barandika, Francisco Ruiz-Fons, Ione Ortega-Araiztegi, Isabel Jado, Ana Hurtado, Ana Luisa García-Pérez

**Affiliations:** 1 NEIKER - Instituto Vasco de Investigación y Desarrollo Agrario, Animal Health Department, Bizkaia Science and Technology Park 812L, 48160 Derio, Biscay Spain; 2 Animal Health and Biotechnology Group (SaBio), Instituto de Investigación en Recursos Cinegéticos IREC (CSIC-UCLM-JCCM), Ciudad Real, Spain; 3 Centro Nacional de Microbiología - Instituto de Salud Carlos III, Majadahonda, Madrid, Spain

**Keywords:** *Coxiella burnetii*, Dairy sheep, Bulk tank milk, SNP genotyping

## Abstract

Bulk tank milk (BTM) samples were collected from 81 sheep flocks in the Basque Country, Spain, in 2015 and were analysed for antibodies against *Coxiella burnetii* by ELISA and for *C. burnetii* DNA by real-time PCR. Thirty-two percent of the flocks had BTM antibodies against *C. burnetii*. Presence of *C. burnetii* DNA in BTM was detected in 23% of the flocks, suggesting recent *C. burnetii* infections. Retrospective data of BTM samples obtained from 154 sheep flocks investigated in 2005 in the same geographic area were compiled to assess temporal changes in *C. burnetii* infection. The overall percentage of infected sheep flocks did not significantly change after the 10-year period. Among the 46 flocks sampled in both periods, 11 flocks that were negative in 2005 were positive in 2015, 18 maintained their initial status (positive or negative), and 17 positive flocks were negative in 2015. These findings indicate that *C. burnetii* infection is a dynamic process in dairy sheep in northern Spain. Single nucleotide polymorphism (SNP) genotyping of positive samples identified three genotypes, SNP1 being the most prevalent in 2015 and SNP8 in 2005; SNP4 was only detected once in 2005. These results suggest possible changes in the pattern of genotype infection over time.

## Findings

Q fever is a worldwide distributed zoonosis caused by *Coxiella burnetii*. Domestic ruminants are the main reservoir and source of infection for humans [1]. *C. burnetii* can produce abortion in domestic ruminants such as cattle, sheep and goats [2]. To prevent Q fever outbreaks both in animals and in people, it is important to monitor the presence and prevalence of *C. burnetii* in livestock farms to establish effective control measures. Serological tests on bulk tank milk (BTM) samples are very useful for the epidemiological surveillance of some infections in dairy livestock. In the case of *C. burnetii*, detection of antibodies in BTM is indicative of previous contact of the herd with the pathogen, whereas detection of the pathogen would be indicative of a current and active infection. The evolution of infection can be also monitored by periodic analyses of BTM samples, as shown for dairy cattle [3] and goats [4]. *C. burnetii* DNA obtained from positive BTM samples can be genotyped to determine strains present [5, 6].

In the Basque Country, northern Spain, dairy sheep attains both the highest *C. burnetii* flock seroprevalence (74%) and the highest within-flock seroprevalence (11.8%) compared to other domestic ruminants [7], suggesting that sheep could be the main reservoir of infection in this area. Lambing in Latxa sheep flocks occurs once a year with an early peak between November and February for ewes in their second and subsequent lactations and a second peak in March–April for yearlings. Lambing is followed by a milking period of 3–4 months. Once milking finishes, many flocks have access to communal mountain pastures during summer and autumn where they widely interact with other grazing sheep flocks, goats, cattle and wildlife. According to the last census (2015) there were 259,569 Latxa breed sheep in northern Spain, showing a reduction of 30% in the last 10 years (354,445 sheep in 2005) (http://www.eustat.eus/banku/id_4017/indexLista.html). A survey carried out in 2005 in 154 dairy sheep flocks indicated that *C. burnetii* was actively circulating in the region [8]. In this context, 10 years later, this study was aimed at (i) identifying changes in the prevalence of *C. burnetii* in dairy sheep after a period of 10 years in the area, and (ii) characterizing the genotypes infecting dairy sheep in the region in both time points to evaluate changes over time. No compulsory control actions against Q fever had been taken during the 10-year period.

BTM samples were collected from 81 sheep farms in March–April 2015, when both, ewes and yearlings, were being milked. Serological analyses were performed by an enzyme-linked immunosorbent assay (ELISA) (PrioCHECK™ Ruminant Q Fever Ab Plate ELISA Kit, Thermo Fisher Scientific, USA) according to the manufacturer’s instructions. DNA was extracted using the QIAmp DNA Blood Mini Kit (Qiagen Hilden, Germany), with modifications already described [3], and presence of *C. burnetii* DNA was investigated by real-time polymerase chain reaction (rt-PCR) amplification targeting the transposon-like repetitive region IS*1111* of *C. burnetii* [9], including a commercial internal amplification control (TaqMan^®^ Exogenous Internal Positive Control, Thermo Fisher Scientific) to monitor for PCR inhibitors.

The percentage of flocks with antibodies against *C. burnetii* in BTM samples was 32.1% (26/81) and the percentage of flocks with *C. burnetii* DNA in milk was 23.5% (19/81). Three flocks were BTM negative by ELISA but low levels of bacterial shedding were detected by rt-PCR (Ct 33–35).

In order to assess changes in *C. burnetii* infection after a 10-year period, BTM ELISA and PCR results from 154 sheep flocks sampled in March–April 2005 [8, 10] were compiled and Chi-square tests were used to compare infection prevalence (2015 vs. 2005). The ELISA test used in both studies was the same (commercialized by LSI, France in 2005), but the PCR method differed (conventional PCR was used in 2005, rt-PCR in 2015). The number of sheep flocks surveyed in both studies represented 30% of the professionally managed flocks with over 100 reproductive ewes in the study region. ELISA and PCR results obtained in 2015 did not significantly differ from those obtained in 2005 (Table 1). However, a slight decrease in the prevalence of flocks with antibodies against *C. burnetii* (40.3% in 2005 vs. 32.1% in 2015; χ^2^ = 1.51, df = 1, P > 0.05) and a slight increase in the percentage of flocks with *C. burnetii* DNA in the BTM were observed (22.1% in 2005 vs. 23.5% in 2015; χ^2^ = 0.06, df = 1, P > 0.05).

**Table 1:** Percentage of *Coxiella burnetii* positive flocks and SNP genotypes identified in bulk tank milk samples collected in 2005 and 2015 in northern Spain

BTM analyses					Single nucleotide polymorphism (SNP)				
Year	N	ELISA	PCR	Ref.	N	SNP1	SNP4	SNP8	Ref.
		Positive (%)	Positive (%)			Positive (%)	Positive (%)	Positive (%)	
2005	154	62 (40.3%)	34 (22.1%)	[8, 10]	16	3 (18.8%)	1 (6.3%)	12 (75.0%)	This study
2015	81	26 (32.1%)	19 (23.5%)	This study	12	10 (83.3%)	0 (0.0%)	2 (16.7%)	This study

Comparison of both series of data identified 46 flocks which were sampled in both surveys. Again, non-significant differences similar to those described for the whole dataset of farms were observed when considering seroprevalence (43.5% in 2005 vs. 37.0% in 2015; χ^2^ = 0.41, df = 1, P > 0.05) or bacterial (DNA) shedding (21.7% in 2005 vs. 28.3% in 2015; χ^2^ = 0.47, df = 1, P > 0.05). The different molecular techniques used in both periods, conventional PCR vs. rt-PCR, the latter being more sensitive [11], could have contributed to the slight but non-significant increase in shedding. Unfortunately, the small amount of DNA available from BTM samples collected in 2005 prevented us from reanalysing them with rt-PCR. Changes in the *C. burnetii* status of some flocks were observed between samplings when considering a flock as “negative” when BTM was negative by ELISA and PCR, and as “positive” when positive by ELISA or PCR (Table 2). Thus, 11 flocks that were negative in 2005 were positive in 2015, 18 maintained their initial status (positive or negative), and 17 positive flocks were negative in 2015. Interestingly, 8 of the 25 positive flocks in 2005 were still positive in 2015. Had vaccination been implemented, the prevalence of *C. burnetii* might have decreased significantly as happened in The Netherlands [4]. In the region of the current study, an inactivated vaccine has only been used at an individual basis by a scarce number of sheep breeders and no specific collective control actions were implemented from 2015 to 2015. The results may suggest that infectious stages of *C. burnetii* persisted in the farm environments during this period in the 8 flocks that remained positive. Alternatively, reinfections could have also occurred during this 10-year period e.g., due purchase of infected animals, contact with other infected flocks, wildlife, etc., with an unnoticed period of infection clearance between samplings.

**Table 2:** *Coxiella burnetii* infection status in 2005 and 2015 of the 46 sheep flocks from northern Spain analysed in both periods

Numbers of examined sheep flocks	*C. burnetii* status in 2005	*C. burnetii* status in 2015
11	Negative	Positive
10	Negative	Negative
17	Positive	Negative
8	Positive	Positive

**Fig. 1:**
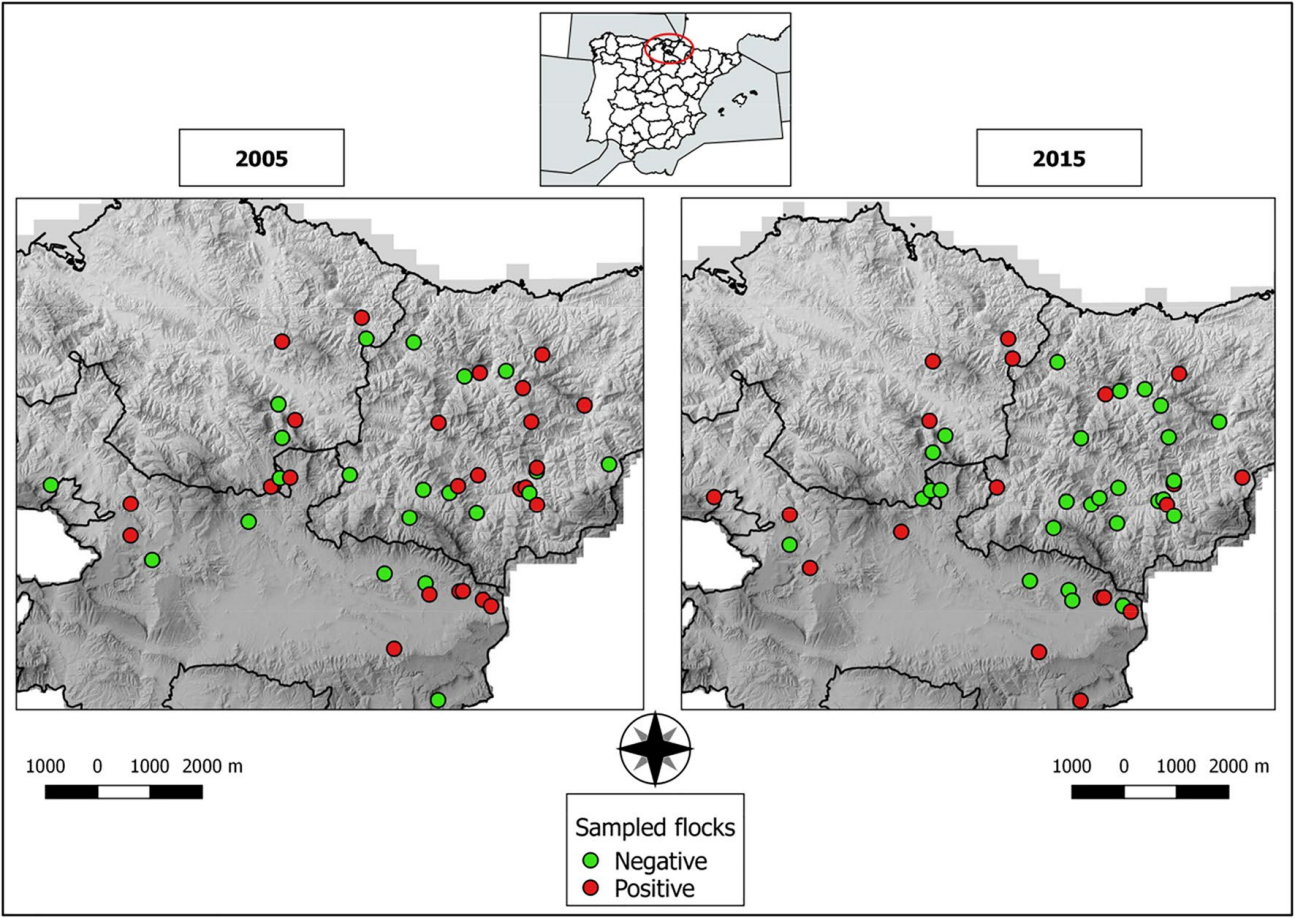
Spatial geographical location of 46 sheep farms in northern Spain sampled in 2005 and 2015 and their *Coxiella burnetii* status. Negative flocks (ELISA and PCR negative on bulk tank milk) are represented as green dots, where as positive flocks (ELISA and/or PCR BTM-positive) are shown as red dots

Geographical coordinates of the 46 farms sampled in 2005 and 2015 were recorded using a global positioning system (GPS) device. Data on Q-fever status of each flock (positive/negative) were geographically represented using QGIS Las Palmas 2.18.16 Geographical Information System to visualize changes in the spatial distribution of *C. burnetii* overtime (Fig. 1). Considering that *C. burnetii* can be dispersed by the wind, flocks in the vicinity of a positive flock would be expected to be also positive. However, in 2005, negative and positive farms were homogeneously located throughout the sampled territory. Conversely, in 2015, infection seemed to have cleared in some flocks from the eastern part of the region. Mapping the epidemiological status of *C. burnetii* in the studied flocks showed that in some areas the infection seemed to clear out without specific control measures while in other areas it seemed to persist for years. The analysis of more than one BTM sample per lactation period and additional intermediate controls during the 10-year time-frame should have been performed to get more accurate data on *C. burnetii* persistence over time. However, this was a first approach using this methodology that will be further developed for the evaluation of the efficacy of vaccination-based control measures that will be soon implemented in the Basque Country, Spain.

A subset of rt-PCR positive samples with a Ct < 31 was selected and genotyped by single nucleotide polymorphism (SNP) analysis, implementing the 10 SNP determination already described [12], a highly discriminatory technique that has demonstrated to be valuable for direct genotyping of field samples with low bacterial burdens, such as milk samples [12]. A total of 28 rt-PCR positive samples were genotyped, 16 from 2005 and 12 from 2015, all from different flocks. Three SNP genotypes were identified: SNP1 and SNP8, found both in 2005 and 2015, and SNP4, found only once in 2005 (Table 1). Genotype SNP1 has been detected in goats, sheep, or cattle in several countries such as France, Belgium and The Netherlands [5, 12, 13], and in human patients in the Q fever outbreak from the Netherlands [12]. SNP8 has been described in human infections in Italy and Slovakia, and has been also found in ticks from Russia, and in ticks and small mammals from Slovakia [12]. Recently, we found SNP1 (MST13) and SNP8 (MST18) in Spanish goat farms identified as the most probable sources of two outbreaks of Q fever infections in humans experiencing fever and pneumonia [14, 15]. Therefore, sheep carrying *C. burnetii* genotypes SNP1 and SNP8 could also pose a risk for human infections in the study area. Comparison of frequencies of genotypes by Chi-square tests revealed that distribution of SNP genotypes changed significantly in the two periods. Hence, whereas in 2005 SNP8 was the predominant type (12/16), in 2015 SNP1 was the most prevalent (10/12) (Fisher exact test, P < 0.01), indicating changes in the pattern of genotype infection over time, possibly due to the infection by co-circulating *C. burnetii* strains, and/or the evolution of previously detected strains [16]. In fact, the presence of multiple *C. burnetii* strains within a single sheep flock has been reported previously [17]. Also, SNP1 dominance in 2015 might be the result of a recent adaptation of this genotype in sheep and a rapid dispersal within the sheep population. However, this hypothesis should be confirmed in further studies. Interestingly, SNP4, recovered from human blood in Slovakia [12], was also identified in one flock in 2005 and was not detected thereafter. These results show the importance of understanding the natural dynamics of this zoonotic pathogen in its major reservoirs to efficiently prevent the negative effects caused by Q fever to animal production and public health.

In conclusion, *C. burnetii* infection presents a dynamic pattern in the studied sheep population. However, the observed trend indicated a stability in the overall percentage of infected sheep flocks in a region where no collective intervention measures have been yet implemented. The analysis of BTM samples in dairy ruminants is an easy strategy to identify infected flocks. Antibody levels in BTM are consistent with findings in serum of dairy ewes over time [18]. In addition, PCR analysis of BTM allows identification of animal shedders in the flock [8], but only if BTM samples are collected soon after the start of the milking period of ewes and yearlings since *C. burnetii* shedding through milk in small ruminants is shorter compared to other excretion routes [14, 18].

## Abbreviations

BTM: bulk tank milk
Ct: cycle threshold
ELISA: enzyme-linked immunosorbent assay
GPS: global positioning system
MST: multispacer sequence typing
rt-PCR: real time polymerase chain reaction
SNP: single nucleotide polymorphism
